# Mitral Annulus Disjunction: A Comprehensive Cardiovascular Magnetic Resonance Phenotype and Clinical Outcomes Study

**DOI:** 10.1002/jmri.29524

**Published:** 2024-07-09

**Authors:** Pedro Custódio, Diana de Campos, Ana Rita Moura, Hunain Shiwani, Konstantinos Savvatis, George Joy, Pier D. Lambiase, James C. Moon, Mohammed Y. Khanji, João B. Augusto, Luís R. Lopes

**Affiliations:** ^1^ St Bartholomew's Hospital London UK; ^2^ Hospital Vila Franca de Xira Vila Franca de Xira Portugal; ^3^ Centro Hospitalar Universitário de Coimbra Coimbra Portugal; ^4^ Hospital Distrital de Santarém Santarém Portugal; ^5^ Institute of Cardiovascular Science University College London London UK; ^6^ Queen Mary University of London London UK; ^7^ NIHR University College London Hospitals Biomedical Research Centre London UK; ^8^ Cardiology Department Hospital Fernando da Fonseca Amadora Portugal

**Keywords:** cardiac magnetic resonance, mitral annulus disjunction, ventricular arrhythmia, sudden cardiac death, mitral prolapse, systolic expansion

## Abstract

**Background:**

Clinical importance of mitral annulus disjunction (MAD) is not well established.

**Purpose:**

Characterize a population of MAD all‐comers diagnosed by cardiac magnetic resonance imaging (MRI).

**Study Type:**

Retrospective.

**Population:**

MAD confirmed in 222 patients, age of 49.2 ± 19.3 years, 126 (56.8%) males.

**Field Strength/Sequence:**

1.5 T and 3 T/steady‐state free precession and inversion recovery.

**Assessment:**

Clinical history, outcomes, imaging, and arrhythmia data. MAD defined as a separation ≥2 mm between left ventricular myocardium and mitral annulus. Presence and pattern of late gadolinium enhancement (LGE) were analyzed. LGE in the papillary muscles and adjacent to MAD were identified as MAD related. Ventricular arrhythmias (VA) were grouped into non‐sustained ventricular arrhythmias (NSVA) or sustained. Cardiovascular death assessed.

**Statistical Tests:**

Differences between baseline characteristics were compared. Univariate regression was used to investigate possible associations between ventricular arrhythmia and cardiovascular death with characteristics associated with the severity of MAD. A multivariable logistic regression included significant variables from the univariate analysis and was performed for MAD‐related and global LGE.

**Results:**

MAD extent 5.0 ± 2.6 mm. MV annulus expanded during systole for MAD ≥6 mm. Systolic expansion associated with prolapse, billowing, and curling. LGE present in 82 patients (36.9%). Twenty‐three patients (10.4%) showed MAD‐related LGE by three different observers. No association of LGE with MAD extent (*P* = 0.545) noted. Follow‐up 4.1 ± 2.4 years. No sustained VA observed. In univariable analysis, NSVA was more prevalent in patients with MAD ≥6 mm (33.3% vs. 9.9%), but this was attenuated on multivariate analysis (*P* = 0.054). The presence of NSVA was associated with global LGE but not MAD‐related LGE in isolation (*P* = 0.750). Three patients died of cardiovascular causes (1.4%) and none had MAD‐related LGE. None died of sudden cardiac arrest.

**Conclusion:**

In patients referred for cardiac MRI, mitral valve dysfunction was associated with MAD severity. Scar was not related to the extent of MAD, but associated with NSVA. The risk of sustained arrhythmias and cardiovascular death was low in this population.

**Evidence Level:**

4

**Technical Efficacy:**

Stage 2

Mitral annulus disjunction (MAD) is characterized by dissociation between the fibrous structure of the mitral valve and the ventricular myocardial crest, with atrial displacement of the valvular apparatus and a clear nonmuscular separation between the two structures.^1^, ^2^, ^3^, ^4^ The exact extent of disjunction required to define this condition varies according to different authors and methodologies, ranging from 1 mm to more conservative definitions requiring more than 2 mm.^5^, ^6^, ^7^

The association between MAD and mitral valve prolapse has been described and characterized in different studies, the vast majority by echocardiography.^8^, ^9^, ^10^, ^11^ The awareness of MAD and the advent of widely accessible cardiac magnetic resonance imaging (MRI) allowed the recognition of smaller separations between the myocardial wall and the mitral annulus, thus increasing the number of diagnoses.

Initial reports have suggested that mitral prolapse and/or MAD might be associated with ventricular tachycardia and sudden cardiac death.^11^, ^12^, ^13^, ^14^, ^15^ However, in some reports, all patients had VT or aborted cardiac arrest prior to their inclusion in the study, which might have introduced selection bias.^14^, ^16^ Recent evidence indicates that MAD might be a common finding in the general population, and thus prognostic significance is questionable.^7^

The aim of this study was to characterize MAD using cardiac MRI in a non‐selected population, with comprehensive assessment of 1) morphology of the mitral valve apparatus, including scar in areas related to MAD, and 2) clinical outcomes, including ventricular arrhythmias (VA) and cardiovascular death.

## Methods

### Study Population and Data Collection

**FIGURE 1:**
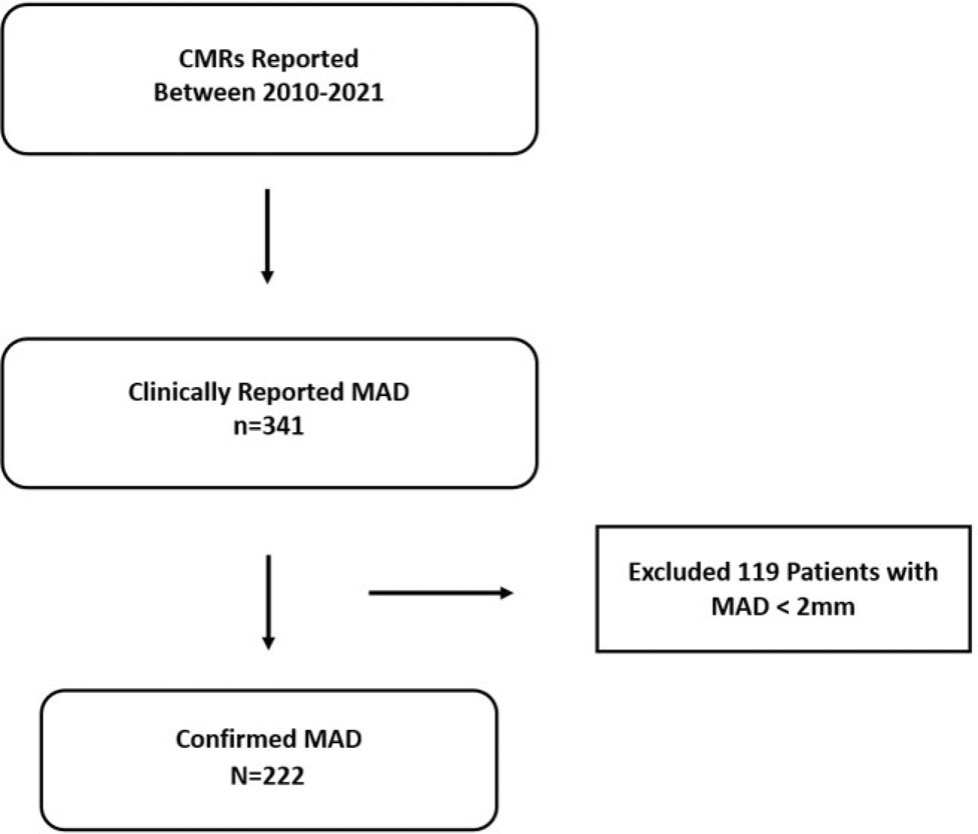
Study flow‐chart. CMR = cardiovascular magnetic resonance; MAD = mitral annulus disjunction.

Single‐center retrospective study from an institutional registry. Patients with MAD were identified from a database of patients who underwent cardiac MRI. The study was approved by the ethics committee (IRAS: 294495). Consecutive patients between January 2010 and July 2022 were identified, with a total of 52,188 scans evaluated. Patients reported as having MAD in cardiac MRI, irrespective of the reason to perform this exam, were singled out (Fig. 1). Images of the patients reported as having MAD were reviewed by three observers (LL, 10 years of experience with European Association of Cardiovascular Imaging [EACVI] level 3 certification in cardiac MRI; PC, 2 years of experience, EACVI level 2; DC, 2 years of experience, EACVI level 2) blinded to clinical data, and MAD was confirmed using a diagnostic criterion of at least 2 mm separation between the mitral annulus and the LV myocardium.^5^, ^6^, ^7^

During the study period, 341 patients were initially reported as having MAD. After revision of those scans, and exclusion of pseudo‐MAD and MAD <2 mm, 222 patients were included (Fig. 1). All images were deemed analyzable by the readers. Mean age was 49.2 ± 19.3 years with 126 (56.8%) males. Characteristics of the study population are detailed in Table 1. Clinical data were collected blinded to the imaging data, including past medical history, biometric evaluation, Holter records, and follow‐up until December 2022.

**TABLE 1:** Baseline Characteristics

	Patients With MAD (N = 222)
Demographics	
Age, years (mean ± SD)	49.2 ± 19.3
Male, N (%)	126 (56.8)
BSA (Du Bois Formula), m^2^ (mean ± SD)	1.87 ± 0.36
Cardiac MRI	
LV ejection fraction, % (mean ± SD)	60.3 ± 8.6
LVEDV, mL (mean ± SD)	162.1 ± 48.8
LVM, g (mean ± SD)	116.2 ± 47.8
RVEDV, mL (mean ± SD)	155.6 ± 43.4
Global LV LGE, N (%)	82 (36.9%)
MAD‐related LGE, N (%)	32 (14.4%)
Past medical history	
*Diabetes mellitus*, N (%)	19 (8.6%)
Hypertension, N (%)	62 (27.9%)
Current or previous smoker, N (%)	18 (8.1%)
Known heart failure, N (%)	64 (28.8%)
Atrial fibrillation, N (%)	17 (7.7%)
Valvular heart disease, N (%)	13 (5.9%)
History of ventricular fibrillation or cardiac arrest, N (%)	2 (0.9%)
Family history of SCD, N (%)	21 (9.4%)
Symptoms	
Previous syncope, N (%)	20 (9%)
Chest pain, N (%)	32 (14.4%)
Shortness of breath, N (%)	66 (30%)

Values are mean ± SD or percentages. BSA = body surface area; LV = left ventricle; LVEDV = left ventricle end‐diastolic volume; LVM = left ventricular mass; RVEDV = right ventricle end‐diastolic volume; SCD = sudden cardiac death.

### Cardiac MRI Acquisition and Analysis

Cardiac MRI scans were conducted using 1.5 T and 3 T scanners (Aera and Prisma models, respectively, Siemens Healthineers, Germany). Pre‐contrast breath‐held steady‐state free precession sequences were used to acquire cine images in standard long and short axis views with a frame rate of 30 per second. Late gadolinium enhancement (LGE) images (long and consecutive short axis slices) were acquired 10 minutes following a bolus administration of 0.1 mmol/kg gadolinium contrast agent (Gadoterate meglumine, Dotarem, Guerbet S.A., France) using a phase sensitive inversion recovery sequence.

All images were analyzed using CVI42 software (Circle Cardiovascular Imaging Inc., Calgary, Canada, version 5.11). Morphological features were assessed, including ventricular volumes and mass, right and left ventricular ejection fraction, mitral annular plane systolic excursion (MAPSE), MV annulus diameters in systole and diastole, prolapse in the anterior and posterior leaflets, leaflets length, billowing, curling, MAD extension (longitudinally and circumferentially), myocardial wall thickness adjacent to the MAD, global LGE (LGE in any left ventricle wall segment) and MAD‐related LGE (LGE in the papillary muscles and/or in the myocardial segments adjacent to the MAD—eg, if a patient with MAD in the inferolateral segment had LGE in the adjacent basal inferolateral myocardium).

MAD was defined as having a separation ≥2 mm between the LV myocardium and the mitral annulus.^17^ MAD circumferential extension was evaluated based on the number of myocardial wall segments that were affected, with a maximum of four segments (anterior, lateral, inferolateral, and/or inferior). MV prolapse was defined as the most atrial part of the coaptation line being >2 mm into the left atrium in systole, when assessed in the 3‐chamber view.^18^ Mitral valve billowing was defined as displacement >2 mm of any leaflet tissue into the LA in systole.^19^ Curling was defined as a paradoxical systolic motion of the posterior mitral ring on the adjacent myocardium.^2^

### Primary Outcome

The primary outcomes were cardiovascular mortality (heart failure, coronary heart disease [CHD], sudden cardiac death) and ventricular arrhythmia. Ventricular tachycardias (VTs) were divided into sustained (i.e., lasting more than 30 seconds) and non‐sustained. Non‐sustained ventricular arrhythmias (NSVA) were defined as non‐sustained ventricular tachycardia (NSVT; ventricular tachycardia >120 bpm for <30 seconds) and/or >10% ventricular ectopics. Events that occurred prior to the cardiac MRI scan were excluded.

### Statistical Analysis

Results were reported as mean ± SD or median with interquartile range. Differences between baseline characteristics were compared using chi‐square tests with Fisher adjustment, Wilcoxon test, or student *t* test, as appropriate. Histogram analysis and Kolmogorov–Smirnov test were used to test for normality. Mitral valve features were assessed—systolic and diastolic annular sizes, anterior and posterior leaflet structure, prolapse, billowing, and curling. Mitral valve disjunction was characterized according to longitudinal and circumferential extent. Univariate regression was used to investigate possible associations between ventricular arrhythmia and cardiovascular death with characteristics expected to be associated with the severity of MAD, based on previous studies,^2^, ^4^, ^5^, ^6^, ^7^, ^8^, ^13^, ^16^ namely sex, extension of MAD (longitudinal and circumferential), prolapse of the MV cusps, curling, billowing, posterior wall involvement and LGE. A multivariable logistic regression was then performed, using the “enter” method, including significant variables from the univariate analysis and others deemed clinically relevant by the researchers. This multivariable regression was performed using sequentially two different concepts of LGE: first, we included LGE expected to be related to MAD (papillary muscles and adjacent myocardium); second, global LV LGE. A bilateral *P*‐value <0.05 was considered statistically significant. Data were analyzed using SPSS® software (version 24, IBM® Armonk, N.Y., USA).

## Results

### Baseline Characteristics

The indications to perform cardiac MRI are depicted in Table S1 in the Supplemental Material, with the assessment of ventricular size and function being the most common (N = 92, 41.4%). In 32 patients (14.4%), the exam was requested due to coronary artery disease and 31 patients (14%) had cardiomyopathy. Two patients (0.9%) had previous ventricular fibrillation or cardiac arrest and 4 (1.8%) had sustained VT as the main indication for the scan. A total of 69 (31.1%) patients had reduced EF (<58%) and 58 (26.1%) patients had dilated LV based on the indexed LVEDV per BSA (>98 mL/m^2^)^20^; 13 patients (5.9%) had increased LV mass (>98 g/m^2^).^21^ The indexed RV volume was increased (>94 mL/m^2^)^20^ in 33 patients (14.9%).

### Mitral Valve Apparatus Characterization

**FIGURE 2:**
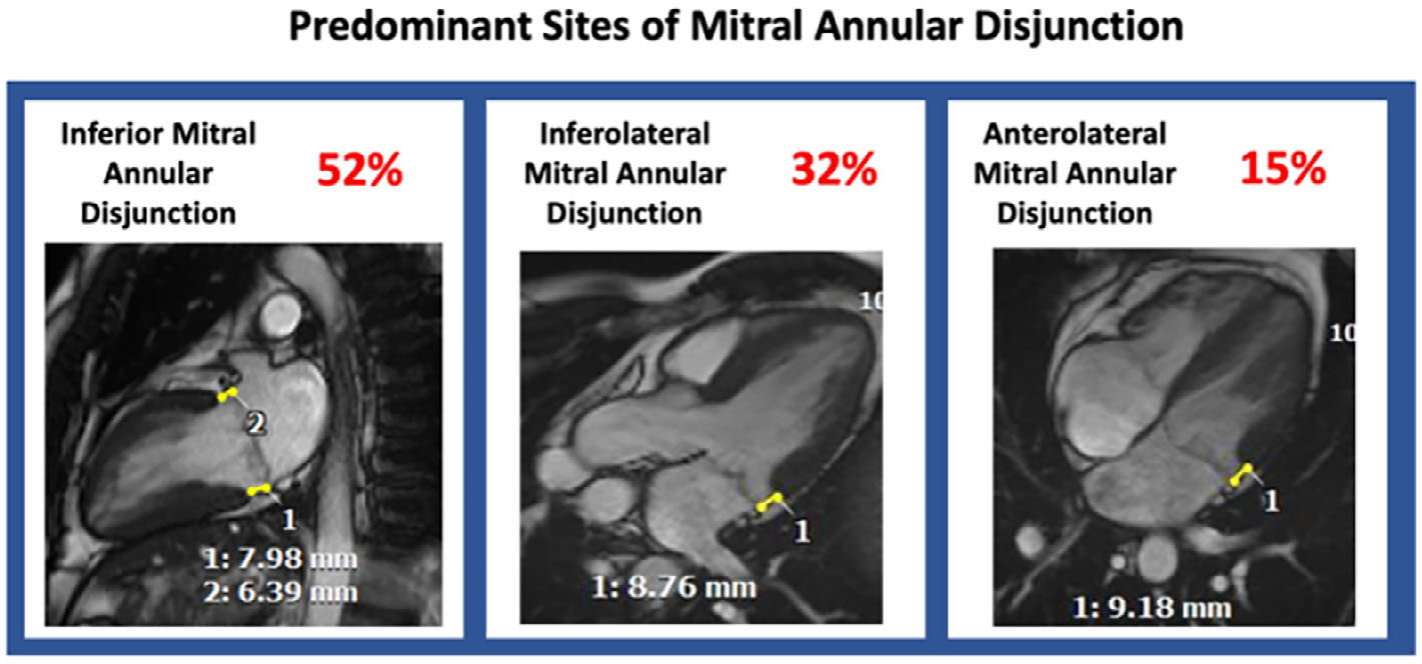
Prevalence of mitral annular disjunction location across the cohort. Inferior (left panel), inferolateral (middle panel), and anterolateral (right panel) segments.

Maximum MAD length was assessed for each patient, ranging from 2 to 17 mm (mean 5 ± 2.6 mm). Most patients had only one segment affected (N = 98, 44.1%). Involvement of more than three segments was less common (N = 18, 8.1%) (mean 1.95 ± 1.001). The wall in which maximal MAD length was most often seen was the inferior (mean 5.1 ± 2.7 mm, 52.3%), followed by inferolateral (mean 5.1 ± 2.4, 32%). More extensive MAD length was associated with progressively increased lateral MAPSE (12.7 ± 2.4 mm for MAD <6 mm vs. 17.1 ± 3.6 for MAD >10 mm). An example of extensive MAD, affecting the anterior, inferior, inferolateral, and anterolateral segments is shown in Fig. 2.

Out of 222 patients, MVP was observed in 48 (21.6%) patients. Twenty‐seven patients had bileaflet involvement, while 13 patients had isolated anterior leaflet prolapse, and 8 isolated posterior leaflet prolapse. Billowing was present in 110 (49.5%) patients, with leaflet involvement as follows: 1) 87 anterior leaflet—average protrusion 5.2 ± 2.4 mm; 2) 74 posterior leaflet—average protrusion 4.5 ± 2.6 mm; 3) 51 had both leaflets protruding (Table 2).

**TABLE 2:** Mitral Valve Characteristics

MV Apparatus	N = 222
Anterior leaflet billowing, N (%)	87 (39.2%)
Posterior leaflet billowing, N (%)	74 (33.3%)
Billowing in both leaflets, N (%)	51 (23%)
MVP, N (%)	48 (21.6%)
Curling, N (%)	32 (14.4%)
Anterior mitral valve leaflet length, mm (mean ± SD)	26.1 ± 5.4
Posterior mitral valve leaflet length, mm (mean ± SD)	16.1 ± 3.8
Lateral MAPSE, mm (mean ± SD)	13.2 ± 4.1
Medial MAPSE, mm (mean ± SD)	9.8 ± 3.5
MV annulus diameter end‐systole, mm (mean ± SD)	37.2 ± 7.7
MV annulus diameter end‐diastole, mm (mean ± SD)	37.6 ± 6.2

Values are mean ± SD or percentages. MVP = mitral valve prolapse; MAPSE = mitral annular planer systolic excursion; TAPSE = tricuspid annular planer systolic excursion.

### Paradoxical Mitral Annulus Expansion

**FIGURE 3:**
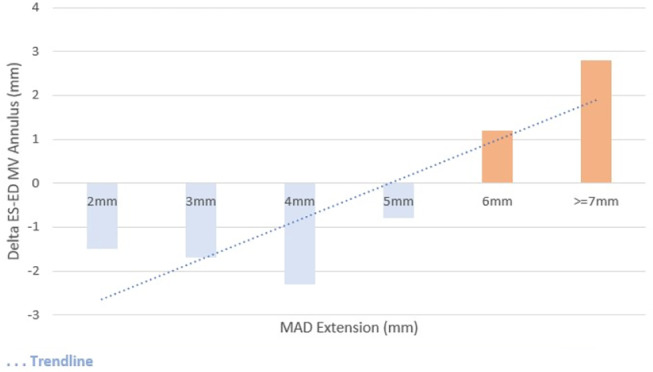
Difference between ES and ED mitral annulus diameter by MAD longitudinal extension. MAD = mitral annulus disjunction; ES = end‐systolic; ED = end‐diastolic; MV = mitral valve.

**FIGURE 4:**
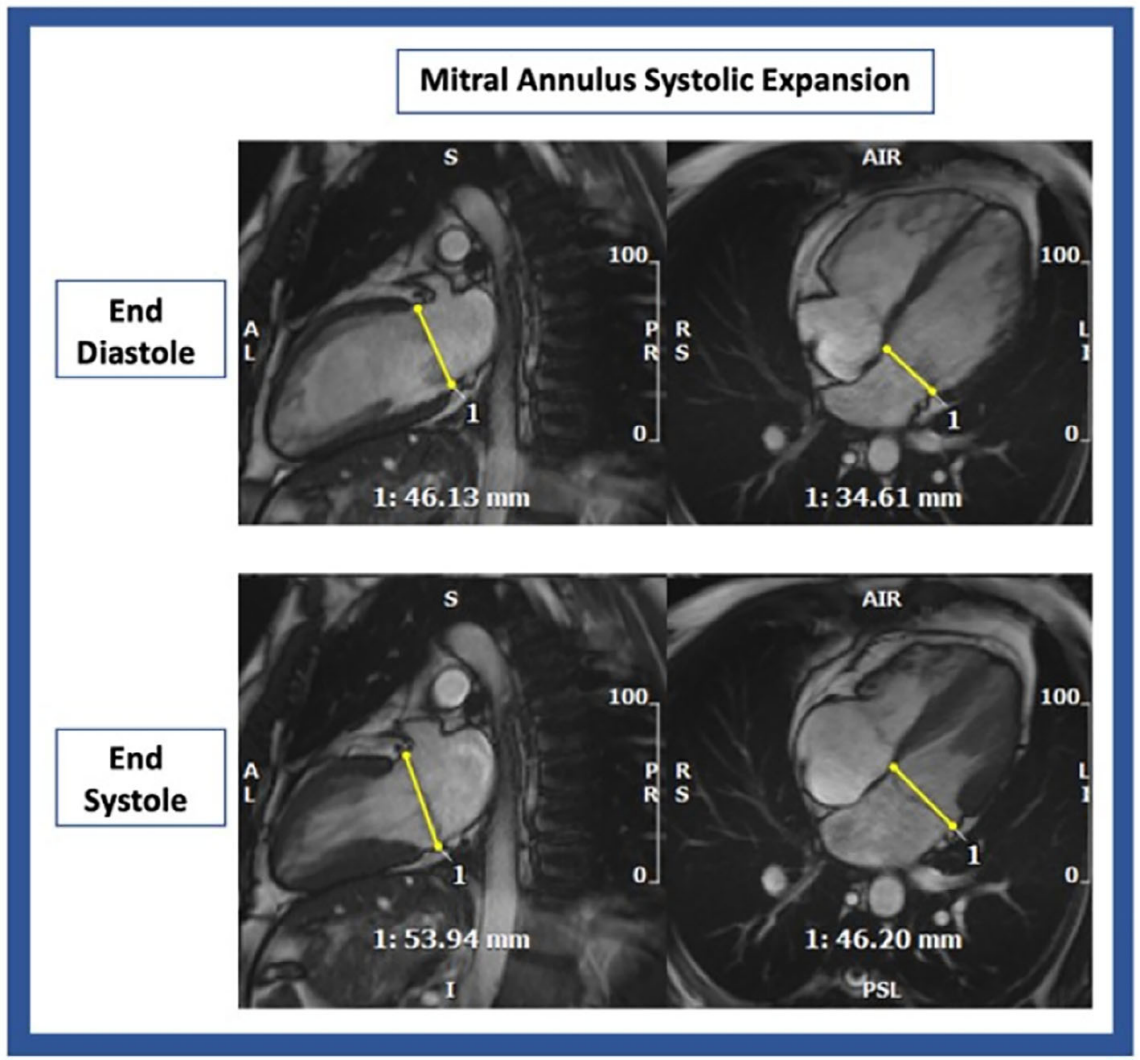
Example of mitral annulus systolic expansion. Top panels—end diastole; bottom panels—end systole.

During systole, the mitral annulus diameter is usually smaller than in diastole, but as the extent of MAD increased, the mitral valve annulus paradoxically expands in systole (on average, from 6.3 ± 2.7 mm MAD onwards), as shown in Fig. 3. An example of extensive MAD associated with mitral annulus expansion is depicted in Fig. 4. Mitral annulus expansion had a significant statistical association with mitral valve prolapse (32.1 vs. 15.5% in patients with mitral annulus contraction), curling (25.9 vs. 7.8%) and higher MAPSE (annular expansion 14.6 ± 4.1 vs. 12.5 ± 4.1 in contraction), as shown in Table 3.

**TABLE 3:** Mitral Valve Comparison Between Systolic Expansion and Contraction

	MV Systolic Expansion (N = 81)	MV Systolic Contraction (N = 141)	*P* Value
MVP, N (%)	26 (32.1%)	22 (15.6%)	**0.003**
Anterior mitral valve leaflet prolapse, N (%)	22 (27.2%)	28 (12.8%)	**0.010**
Extension of anterior mitral valve leaflet prolapse, mm (mean ± SD)	6.2 ± 3.2	5.4 ± 3	**0.006**
Posterior mitral valve leaflet prolapse, N (%)	19 (23.5%)	17 (12.1%)	**0.013**
Extension of posterior mitral valve leaflet prolapse, mm (mean ± SD)	5.1 ± 2.5	4.1 ± 2.6	**0.026**
Curling, N (%)	21 (25.9%)	11 (7.8%)	**<0.001**
Curling extension, mm (mean ± SD)	3.8 ± 1.7	1.6 ± 1.9	**<0.001**
Lateral MAPSE, mm (mean ± SD)	14.6 ± 4.1	12.5 ± 4.1	**<0.001**

Values are mean ± SD or percentages. Values in bold represent *P* <0.05. MV = mitral valve; MVP = mitral valve prolapse; MAPSE = mitral annulus systolic excursion.

### Late Gadolinium Enhancement

Out of 222 patients, 82 (36.9%) showed global LV LGE. The presence of LGE was further analyzed in areas that are typically related to MAD; LGE adjacent to MAD and in the papillary muscles was present in 23 (10.4%) and 15 (6.8%) patients, respectively (six patients had LGE in both locations). The remaining 50 patients (22.5%) had LGE elsewhere. No relation was observed between MAD extension and LGE in MAD‐related areas (*P* = 0.545). MAD >6 mm was not shown to be associated with MAD‐related scar (*P* = 0.472).

### Outcomes

Mean follow‐up post‐MRI was 4.1 ± 2.4 years. During follow‐up, three patients died of cardiovascular causes (heart failure in all cases). No sudden death occurred. Holter records were available for 99 patients, with an average of 67 ± 41.8 hours of monitoring per individual, and a total of 6633 hours of electrocardiographic monitoring. No episode of sustained ventricular tachycardia or ventricular fibrillation was recorded. NSVT was observed in 14 patients (14.1%), three of which were polymorphic. Frequent ventricular ectopy (>10% of the time recorded) was seen in nine patients (9.1%). NSVA were observed in a total of 18 patients (18.2%).

**FIGURE 5:**
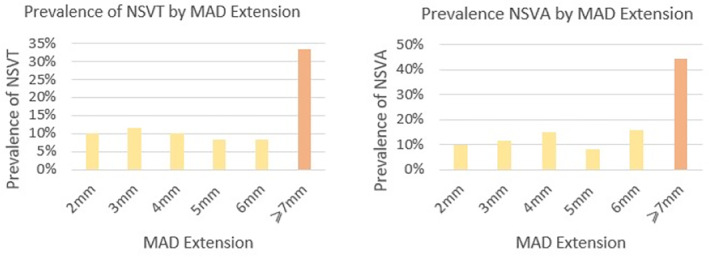
Left panel—prevalence of NSVT stratified by MAD maximum length (mm); right panel—prevalence of NSVA (NSVT + >10% ventricular ectopies) stratified by MAD maximum length (mm). MAD = mitral annulus disjunction; NSVT = non‐sustained ventricular tachycardia; NSVA = non‐sustained ventricular arrhythmia.

By univariate analysis for NSVT, there was a significant association with MAD >6 mm (33.3% vs. 9.9% in patients MAD ≤6 mm), as shown in Fig. 5. The same was observed for all NSVA (44.4% vs. 11.2% in patients MAD ≤6 mm). In univariate analysis, MAD circumferential extension and global LV LGE were also predictors of NSVA, while MAD‐related LGE was not—as depicted in Table 4.

In multivariable analysis, global LV LGE was an independent predictor of non‐sustained VAs (OR 5.808; 95% CI 1.647–20.483), as shown in Table 4. On the other hand, MAD >6 mm showed a tendency, but did not reach statistical significance for an association with NSVA once global LV LGE was taken in consideration (OR 5.070; 95% CI 0.972–16.432; *P* = 0.054). 

**TABLE 4:** Uni and Multivariate Analysis for Non‐Sustained Ventricular Arrythmias

	Univariate			Multivariate		
	OR	95% CI	*P*	OR	95% CI	*P*
Male	0.688	0.246–1.925	0.477			
MV prolapse	1.813	0.556–5.911	0.324			
Billowing	1.528	0.546–4.274	0.419			
MAD >6 mm	5.600	1.788–17.539	**0.003**	5.070	0.972–16.432	0.054
MAD circumferential extension (°)	1.978	1.155–3.386	**0.013**	1.442	0.682–3.049	0.338
Posterior wall involvement	1.922	0.625–5.905	0.254			
Curling	3.034	0.876–10.515	0.08			
LV LGE	3.92	1.328–11.607	**0.013**	5.808	1.647–20.483	**0.006**
MAD‐related LGE	1.255	0.311–5.054	0.75			

Values in bold represent *P* <0.05. OR = odds ratio; CI = confidence interval; MAD = mitral annulus disjunction; MV = mitral valve; LV = left ventricle; LGE = late gadolinium enhancement.

## Discussion

Our main findings are 2‐fold: first, in line with previous studies, we found an increased prevalence of MV prolapse, billowing, and curling with more extensive MAD, namely with MV annulus systolic expansion; second, MAD might be a more benign entity than previously thought—during long‐term follow‐up, no episode of sudden death occurred, nor any episode of VF or sustained VT. NSVA were overall unrelated to the extension of MAD or MAD‐related LGE, but rather to global LV LGE.

The morphological findings of the MV apparatus in patients with MAD appear to be mostly related to the lack of a robust support in the mitral annulus and not due to primary valvular disease. The inferior wall segment was the most frequently involved site, aligned with the findings from Zugwitz et al and Dejgaard et al.^6^, ^7^ Zugwitz et al analyzed cardiac MRI findings from 2646 patients in the UK Biobank imaging study, with MAD defined as present when a separation of at least 1 mm was observed, concluding that MAD is a highly prevalent feature, observed in 76% of the enrolled patients. Dejgaard et al have observed MAD in 116 symptomatic patients by echocardiogram and cardiac MRI, observing a prevalence of NSVT of 22% and aborted cardiac arrest or sustained VT in 12%, although all the latter severe arrhythmic events occurred prior to the inclusion in the trial, which may have led to selection bias. Contrary to what was observed in the two previous reports and by Hutchins et al, the involvement of the inferolateral segment was common in this study population (32%).^20^

The overall prevalence of MV prolapse, billowing, and curling in patients with MAD was similar to a previous publication from Konda et al, but lower than the one reported by Dejgaard et al. Pu‐Wai‐Lee et al has previously described, by 3D echo, the annulo‐ventricular decoupling phenomenon in patients with MAD.^3^, ^11^ In this population, paradoxical MV annulus systolic expansion was mostly observed in patients with more extensive MAD and was a predictor of MV prolapse, billowing, and curling. Specifically, MV annulus systolic expansion appears to be mostly seen in patients with MAD extension >6 mm, while seldomly present in patients with milder phenotypic features.

The absence of a clear association between the extent of MAD and the presence of scar in the adjacent myocardium or papillary muscles was also unexpected. Even in patients with MAD >6 mm, this entity was not a predictor of scar in the areas that could be subject to higher shear stress. This finding disputes the reported causality of MAD‐related scar in previous studies including Marra et al, where MAD was suggested to lead to scarring of the papillary muscles and adjacent myocardium due to mechanical stretch caused by the excessive mobility of the mitral leaflets.^2^

Interestingly, with larger MAD extension, an increase in MAPSE was observed. This seems to be related to hypermobility of the MV annulus and not an increase in myocardial contractility. Thus, MAPSE is likely an unreliable measurement of LV longitudinal systolic function in patients with MAD.

The overall prevalence of VA was slightly lower compared to the publication from Dejgaard et al, but aligned with their prospective findings was mostly accounted by NSVT and frequent isolated ventricular ectopies, instead of sustained VT or VF.^6^ More recently, Esseayagh et al have reported that MAD is frequently observed in MVP and closely related to increased arrhythmic events, despite not being associated with excess mortality in the first 10 years after diagnosis.^6^, ^8^ Studies have suggested that MAD might be an arrhythmogenic entity independently of MVP. The work from Groeneveld et al have reported that the prevalence of inferolateral MAD and MVP was higher in patients with idiopathic VF than in healthy controls.^4^, ^5^ As such, we were intrigued by the absence of sustained arrhythmic episodes in this study despite extensive monitoring. When we consider that our patients were selected from a population with multiple indications to perform a cardiac MRI, and thus likely to have higher prevalence of other cardiac pathologies than the general population, these findings are reassuring about the likely benign nature of this entity in most patients.

The previously published markers of worse arrhythmic outcomes were: involvement of the inferolateral segment, bileaflet MV prolapse, LGE in the papillary muscles, and adjacent myocardium.^12^, ^13^, ^14^, ^16^ Out of the previously described markers of MAD malignancy, only the overall presence of LGE had predictive value for non‐sustained VA, with MAD‐related LGE not having added predictive value. This evidence seems to point to the fact that the risk of VA in patients with MAD is probably mostly related to overall scar burden in the myocardium. We hypothesize that differences from previous studies might be partially justified from a selection bias wherein patients with arrhythmogenic conditions were diagnosed with MAD.

The recent work from Stefano Figliozzi et al was also reassuring about the lack of prognostic impact of MAD in patients with MVP without moderate‐to‐severe mitral regurgitation or LV dysfunction.^22^ Our results are in line with their findings, but expand the results to an all‐comers population.

### Study Limitations

Being a single‐center retrospective study based on patients referred to cardiac MRI, we recognize the inherent limitation of selection bias, and risk of confounding variables and reverse causality in our analysis. Given that data comes from a single center, no repeatability or reproducibility could be ascertained. We have tried to minimize this effect by being extensive in our data collection. The prevalence and reporting of MAD are likely underestimated in our sample of patients, given that awareness to this presentation has grown over the last decade and that different cardiac MRI reporters were likely to have different sensitivities for MAD detection. For this reason, no conclusions on the prevalence of this presentation should be ascertained from our work, particularly since we have only revised the scans where MAD was initially reported and not all cardiac MRI scans. We also recognize that limited echocardiography data were available, posing a pitfall in the assessment and characterization of mitral regurgitation associated with MAD and MVP. We also must mention that having Holter monitoring from just 99 patients might provide a selection bias and potentially compromise the perspective of our cohort of patients.

## Conclusion

In patients referred to cardiac MRI, mitral valve dysfunction (prolapse/billowing) is associated with MAD severity. Systolic mitral annulus expansion appears to be a hallmark of patients with extensive MAD and might play an important role in MV dysfunction. The overall risk of sustained VA and cardiovascular death appears to be low in MAD. NSVA was mostly related to the extent of global LV LGE, but not MAD‐related LGE.

## Conflict of Interest

The authors have no conflicts of interest to declare.

## Supporting information


**Figure S1:** Mitral valve assessment.

**Data S1:** Supporting information.
