# Supplementary material for: Mitral Annulus Disjunction: A Comprehensive Cardiovascular Magnetic Resonance Phenotype and Clinical Outcomes Study
Source: J Magn Reson Imaging. 2024 Jul 9;61(3):1368–75. doi: 10.1002/jmri.29524 (PMC11803685; doi:10.1002/jmri.29524)
Supplement: Supplementary file 1 — Figure S1: Mitral valve assessment. [file JMRI-61-1368-s001.docx]

**SUPPLEMENTARY FIGURE 1 – Mitral valve assessment**

**
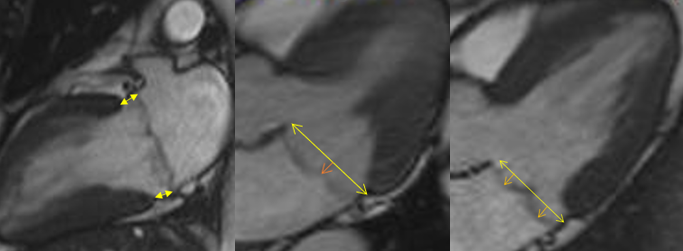
**

Left panel – Measurement of the MAD in the anterior and inferior wall segments (yellow arrow); Mid panel – measurement of the mitral valve prolapse (dark orange arrow); Right panel – Measurement of the billowing of the anterior and posterior mitral valve leaflets (light orange arrows)
