# Supplementary material for: Mitral Annulus Disjunction: A Comprehensive Cardiovascular Magnetic Resonance Phenotype and Clinical Outcomes Study
Source: J Magn Reson Imaging. 2024 Jul 9;61(3):1368–75. doi: 10.1002/jmri.29524 (PMC11803685; doi:10.1002/jmri.29524)
Supplement: Supplementary file 2 — Data S1: Supporting information. [file JMRI-61-1368-s002.docx]

**Supplementary Tables**

**SUPPLEMENTARY TABLE 1 - Main reason to perform Cardiac MRI**

| **Reason for Referral** | **Patients** |
| --- | --- |
|  | **(N= 222)** |
| Ventricular function and volumes assessment, n (%) | 92 (41.4) |
| CAD and ischemia, n (%) | 32 (14.4) |
| Known cardiomyopathy, n (%) | 31 (14) |
| Suspicion of cardiomyopathy (family+/gene+), n (%) | 23 (10.4) |
| Myocarditis, n (%) | 8 (3.6) |
| Valve disease, n (%) | 13 (5.9) |
| AF, n (%) | 4 (1.8) |
| Ventricular ectopy, n (%) | 7 (3.2) |
| VT, n (%) | 4 (1.8) |
| VF and cardiac arrest, n (%) | 2 (0.9) |
| Aortic pathology, n (%) | 3 (1.4) |
| LV thrombus, n (%) | 2 (0.9) |
| Syncope, n (%) | 1 (0.5) |

CAD – coronary artery disease; AF – atrial fibrillation; VT – ventricular tachycardia; VF- ventricular fibrillation, LV – left ventricle.

**SUPPLEMENTARY TABLE 2 – Cardiac MRI sequence used per cardiac structure assessed**

| Cardiac Structure | Sequence Used |
| --- | --- |
|  |  |
| Valves | Breath-held steady-state free precession – 30 frames per second |
| Ventricular size and function | Breath-held steady-state free precession – 30 frames per second |
| Mitral annulus disjunction | Breath-held steady-state free precession – 30 frames per second |
| Myocardial scar | Inversion recovery (late gadolinium enhancement) |
